# Sepsis: network pathophysiology and implications for early diagnosis

**DOI:** 10.1152/ajpregu.00003.2023

**Published:** 2023-03-06

**Authors:** Jaskirat Arora, Asher A. Mendelson, Alison Fox-Robichaud

**Affiliations:** ^1^Department of Medical Sciences, McMaster University, Hamilton, Ontario, Canada; ^2^Section of Critical Care Medicine, Department of Medicine, Rady Faculty of Health Sciences, University of Manitoba, Winnipeg, Manitoba, Canada

**Keywords:** biomarkers, immunothrombosis, innate immunity, neutrophil extracellular traps, platelets

## Abstract

Sepsis, a medical emergency, is the overwhelming host response to infection leading to organ failure. The pathophysiology of this heterogeneous disease includes an inflammatory response that stimulates a complex interaction between endothelial and complements with associated coagulation abnormalities. Despite a more comprehensive understanding of sepsis pathophysiology, there exists a translational gap to improve sepsis diagnosis clinically. Many of the proposed biomarkers to diagnose sepsis lack sufficient specificity and sensitivity to be used in routine clinical practice. There has also been a lack of progress in diagnostic tools due to the focus on the inflammatory pathway. Inflammation and coagulation are known to be linked to the innate immune response. Early immunothrombotic changes could result in the early switch from infection to sepsis and aid in sepsis diagnosis. This review integrates both preclinical and clinical studies that highlight sepsis pathophysiology providing a framework for how the development of immunothrombosis could be used as a starting point to investigate biomarkers for early sepsis diagnosis.

## INTRODUCTION

**Figure 1.**
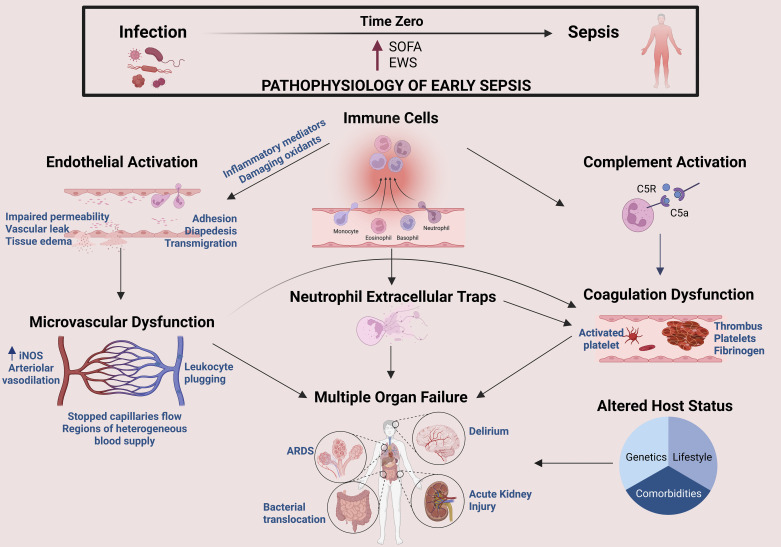
Pathophysiology of sepsis. A schematic outline of the critical switch from infection to sepsis is termed “Time Zero.” The host-defense mediators elicit exaggerated immune cell response stimulating the complement system and collaterally damaging the endothelium and microvasculature. This figure was created with BioRender.com. ARDS, acute respiratory distress syndrome; EWS, early warning score; iNOS, inducible nitric oxide synthase; SOFA, sequential organ failure assessment.

Sepsis, a life-threatening organ dysfunction caused by an exaggerated host response to infection, is one of the leading causes of death worldwide (1). Globally, there are ∼48.9 million sepsis cases, leading to 11 million deaths annually (2, 3). Sepsis is one of the most expensive medical conditions to treat. Before the coronavirus disease 2019 pandemic, sepsis costs were about $1.3 billion/per year in Ontario, Canada, and $27 billion in the United States (2, 3). The average hospital length of stay for sepsis is twice as long as any other fatal condition, and the in-hospital mortality remains as high as 20% (4, 5). Furthermore, sepsis survivors are at an increased risk of death or a reduced health-related quality of life even after discharge from the hospital (6–8). Hence, sepsis is a significant contributor to the global health burden of diseases. Approximately 80% of septic cases begin treatment in the emergency department, and the rest are transferred to the other departments of the hospital (9). There are three major problems with sepsis diagnosis: *1*) the clinical symptoms are not specific to sepsis; *2*) no biomarker has sufficient sensitivity and specificity to identify sepsis due to the complex pathophysiology; and *3*) sepsis is a heterogeneous syndrome with no unifying cause, phenotype, or biological characteristics (10, 11). These challenges require urgent attention, as early diagnosis and treatment are essential for improving sepsis outcomes—ideally within 3 h as outlined by the best practice guidelines (9, 12). This review will describe the pathophysiology of the host response in sepsis within three archetypal biological domains, highlight their complex interplay, and discuss the implication for early diagnosis (Fig. 1).

## IMMUNE SYSTEM DYSREGULATION: INNATE AND ADAPTIVE IMMUNE RESPONSES

**Figure 2.**
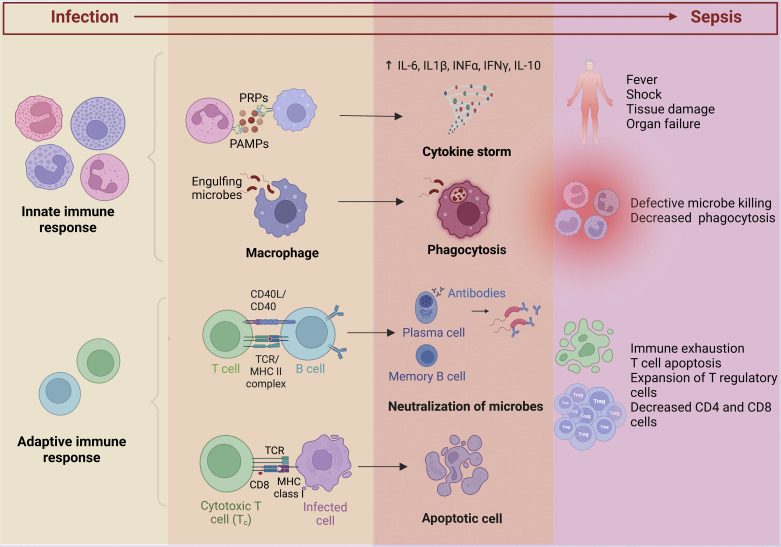
Immune system activation in sepsis. During sepsis, the systemic activation of the immune system results in an inflammatory response characterized by cytokine storm with associated fever, shock, and multiple organ dysfunction. The adaptive immune response produces pathogen-specific antibodies with immunological memory for subsequent exposures to the same antigen. Sepsis-induced immunosuppression causes apoptotic depletion of immune cells, immune exhaustion, and decreased CD4 and CD8 cells. This figure was created with BioRender.com. PAMPS, pathogen-associated molecular patterns; PRP, pattern recognition protein; TCR, T-cell receptor.

Sepsis is an inflammatory host response to infection. The innate immune system is activated in response to pathogens via the binding of pathogen-associated molecular patterns to specific pattern recognition receptors (PRRs) (Fig. 2) (13, 14). Activation of PRRs triggers intracellular signaling that stimulates the activation of transcription factors such as nuclear factor-κB and interferon regulatory factor pathways to release inflammatory cytokines (13). The timeline of the complex immunological alterations in sepsis is poorly understood. Originally, multiple studies demonstrated that the compensatory anti-inflammatory response occurs after hyperinflammation. However, recent studies postulate that an immunoparalysis phase follows the immediate hyperinflammatory mediators (15). The initial cytokine storm causes fever, shock, respiratory failure, and early death due to multiple organ dysfunction (16, 17). It was postulated that sepsis resulted from a “cytokine storm” induced by proinflammatory mediators; recently, it has been shown that anti-inflammatory mediators also accompany the release of proinflammatory cytokines (18). Proinflammatory cytokines stimulate adhesion molecule expression, such as vascular cell adhesion molecule-1 (VCAM-1), in the coronary endothelium, and neutrophils infiltrate the myocardium and reduce cardiomyocyte contractility (19). The adaptive immune response triggered by antigen-presenting cells, dendritic cells, and B and T lymphocytes is slower, in contrast to the innate immune system, and produces pathogen-specific antibodies with immunological memory for enhanced response to subsequent exposures from the same antigen (20). Hence, the course of sepsis pathophysiology involves both a pro- and anti-inflammatory response influencing sepsis progression.

Sepsis-induced immunosuppression, also called “immune exhaustion,” involves the apoptotic depletion of immune cells (21–24). The innate and adaptive immune cells undergo apoptosis contributing to reduced clearance of invading pathogens (24–28). This cell depletion is spanned across all-patient age groups (21, 29). Apoptotic depletion of cells occurs in a greater magnitude among sepsis nonsurvivors than survivors (23, 30–32). Apoptotic depletion of CD4+ T cells results in decreased cytokine production interleukin (IL)-2, IL-12, and interferon-γ (IFN-γ) by the subsets of CD4^+^ T cells, particularly T helper (Th)1 and Th2 (23, 33). T cells can develop a state of functional unresponsiveness referred to as “exhaustion” due to prolonged antigen exposure and altered differentiation of memory T cells (30). There is an increase in T regulatory suppressor cells with concomitant loss of effector T cells, associated with higher sepsis-related mortality (34, 35). Because of the suppression of cell-based immunity, the mortality associated with the late phase of sepsis is due to acquired secondary and opportunistic infections such as *Candida* (36). The immune system stimulates endothelial and contributes to microcirculatory failure that intertwines in the sepsis pathophysiology.

## ENDOTHELIAL AND MICROCIRCULATORY DYSFUNCTION IN SEPSIS

**Figure 3.**
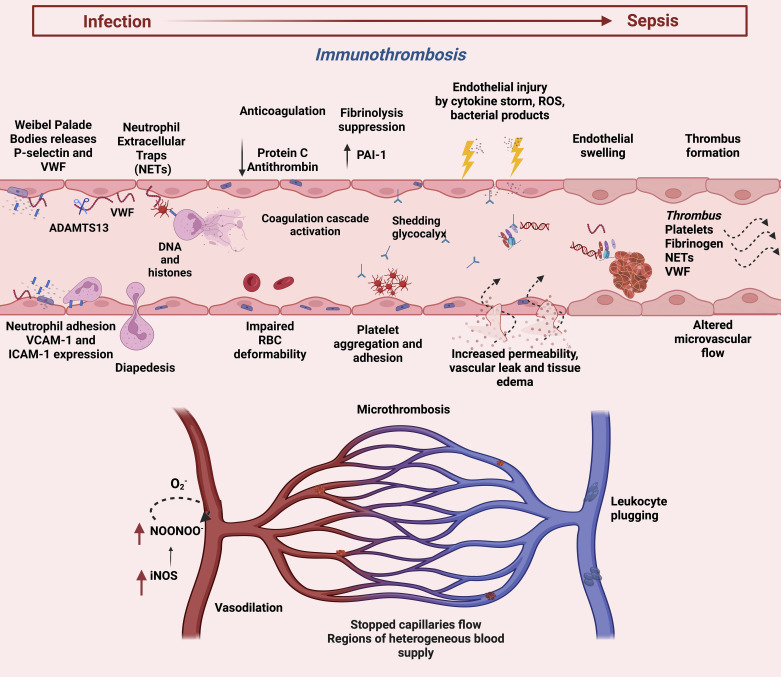
Endothelial activation and coagulation dysfunction in sepsis. Weibel Palade bodies release P-selectin and VWF. ADAMTS13, a metalloprotease, cleaves highly procoagulant VWF multimers into lesser procoagulant forms. Platelets interact with activated neutrophils to induce NETs formation. NETs shift the balance toward excessive coagulation together with downregulation of anticoagulation and antifibrinolysis. The cascade of endothelial injury induces increased vascular permeability with associated coagulation abnormalities, altered microvascular flow, and micro thrombosis. This figure was created with BioRender.com. ADAMTS13, a disintegrin and metalloproteinase with thrombospondin motifs; ICAM-1, intercellular adhesion molecule 1; iNOS, inducible nitric oxide synthase; NETs, neutrophil extracellular traps; PAI-1, plasminogen activator inhibitor-1; VCAM-1, vascular cell adhesion molecule; VWF, von Willebrand factor.

It has been suggested that endothelial cells form a dynamic equilibrium between inflammation, innate immunity, complement, and coagulation to delicate a host response in sepsis (Fig. 3) (37). Microvascular dysfunction in sepsis begins with activation of the endothelium and changes to a proinflammatory phenotype for endothelial cells (ECs) (38). The cytokine storm during hyperinflammation damages the endothelium, causing dysregulated vascular tone and homeostasis, impairing the vascular permeability barrier (38–42). Although ECs share common characteristics, organ-specific features are observed due to the heterogeneity of different microcirculatory beds (43–45). The integrity of the endothelial lining regulated by the endothelial cytoskeleton and glycocalyx is disrupted by the release of reactive oxygen species, inflammatory cytokines, and bacterial endotoxins leading to glycocalyx shedding (46–50). Acute respiratory distress syndrome (ARDS) caused by widespread endothelial barrier dysfunction in the lungs mediated by proinflammatory cytokines with increased pulmonary vascular permeability and diffused pulmonary infiltrates leads to the accumulation of protein-rich fluid predisposing to acute respiratory failure (51). In addition, the glycocalyx layer helps to modulate the leukocyte-endothelial interactions under physiological conditions (46). Glycocalyx shedding enhances leukocyte activation, adhesion, and extravasation by exposing adhesion molecules (such as P-selectin, E-selectin, and intercellular adhesion molecule-1) that facilitate the recruitment of leukocytes and platelets (47–50, 52). Hence, endothelial injury by proinflammation injury induces impaired permeability that subsequently activates the coagulation system, depicting the intertwined sepsis pathophysiology.

Under physiological conditions, the endothelium maintains the balance between coagulation and fibrinolysis to prevent systemic bleeding and clotting, maintaining hemostasis (53). The endothelium synthesizes and expresses molecules that are vital in regulating hemostasis, such as von Willebrand factor (VWF), tissue factor (TF), and plasminogen activator inhibitor type-1 (PAI-1) (54–58). VWF, the main cargo of Weibel palade bodies, mediates platelet adhesion and aggregation (58, 59). Reduced levels of the VWF proteolytic scissor, ADAMTS13 antigen in sepsis promotes formation of small blood clots in the bloodstream and leads to disseminated intravascular coagulation (DIC) (58). P-selectin released by exocytosis with VWF is involved in leukocyte rolling and recruitment in inflammation, making VWF an acute phase response protein (60, 61). Activated endothelial cells and leukocytes express high TF levels stimulating the extrinsic clotting cascade by binding to factor VII, resulting in thrombin generation (62–64). The proteolytic inactivation of tissue factor pathway inhibitor (TFPI) and reduced activation of protein C (PC) facilitates a prothrombotic phenotype (65). The elevated production of PAI-1 by endothelium suppresses the fibrinolytic pathway by inhibiting tissue plasminogen activator (tPA) (66). These defects increase the production of fibrin-rich microvascular clots in sepsis, predisposing the vessels to DIC.

The microcirculation constitutes arterioles, capillaries, and postcapillary venules, involved in the pathophysiological processes in sepsis, including endothelial dysfunction, activation of coagulation, loss of smooth muscle tone reactivity, and disordered leukocyte sequestration (67). The endothelial cells regulate the local distribution of blood flow and oxygen delivery through the release of vasodilators, especially nitric oxide (NO), modulating arteriolar smooth muscle cell tone (68). Inducible nitric oxide synthase (iNOS) is known to be upregulated in sepsis but is heterogeneously expressed in the vascular beds, resulting in both hypo- and overperfusion that contributes to mismatched oxygen delivery (42, 69, 70). RBC velocity and supply rate in capillary networks is reduced in sepsis, as demonstrated by preclinical studies and human studies leading to impaired convective transport of oxygen (71–73). This is exacerbated by decreased functional capillary density and increased stopped-flow capillaries caused by reduced deformability of the RBCs and platelet-fibrin plugging (74, 75). The smooth muscle cells lining arterioles lose their ability to regulate perfusion and tone in sepsis (76). RBCs lose their ability to release vasodilators, impair RBC/O2 signaling, and causing decreased oxygen delivery during sepsis (77). Impaired oxygen delivery to the tissues fails to meet the oxygen demand, ensuing anaerobic glycolysis lactic acidosis.

## COAGULATION AND COMPLEMENT DYSFUNCTION

The coagulation dysfunction in sepsis ranges from mild-subclinical to severe hematological dysfunction presenting as prolonged prothrombin time, increased D-dimer levels, and low platelet counts leading to DIC (78, 79). The etiology of the dysregulation of coagulation in sepsis involves two important innate immune cells: platelets and neutrophils. Stimulated platelets express P-selectin on their surface, facilitating platelet-leukocyte interactions through the receptor P-selectin glycoprotein ligand-1 on leukocytes facilitating cell trafficking and activation (80). Takei et al. (81) discovered neutrophil extracellular traps (NETs) in 1996 as a cell death mechanism different from apoptosis and necrosis. This cell death mechanism has a major prognostic impact on patients with sepsis, similar to apoptotic death (23, 28, 82–84). NETs are composed of extracellular chromatin wrapped around histones and numerous granular proteins (such as elastase and myeloperoxidase) engulfing the invading pathogens (85, 86). Most bacteria are killed readily by neutrophils; however, some bacterial pathogens can circumvent destruction by NETs (86, 87). Peptidyl arginine deiminase-4 (PAD4) citrullinates the histones and relaxes the chromatin releasing cell-free DNA (cfDNA) into the circulation (88). Platelets bind to neutrophils via the platelet Toll-like receptor 4 (TLR4) that stimulates NETs production (89). Similarly, histones during NETs generation can activate platelets via Toll-like 2 (TLR2) and TLR4 receptors (90). Consequently, platelet recruitment leads to NETs formation and clot growth that entraps bacteria in the microvasculature (91–93). Although NETs protect the host by limiting microbial growth and dissemination, excessive NETosis during sepsis can shift the hemostatic balance toward excessive coagulation, promoting thrombus formation (94–97). CfDNA enhances thrombin generation by activating the intrinsic pathway (94). Deoxyribonuclease, known to degrade CfDNA, improves sepsis mortality; however, its absence leads to vascular occlusion and organ damage (98–100). In addition, sepsis is characterized by a drop in platelet count due to their sequestration and consumption by microthrombi (101). The dysregulated immune response leads to systemic activation of blood coagulation to a variable degree inducing microvascular clotting due to massive thrombin and fibrin formation (102). This can manifest as DIC forming thrombi within small and medium vessels, leading to multiorgan failure (102). Therefore, platelets and neutrophils promote innate immune cell responses and procoagulant action. This depicts an interlinked immunothrombotic mechanism in sepsis.

Sepsis downregulates the anticoagulant and fibrinolytic mechanism of the body. NETs augment fibrinolysis, promote the stability of fibrin clots, and inhibit plasminogen activators (95–97). The activated PC is reduced due to the downregulation of endothelial cell protein C receptor and thrombomodulin, decreased production by the liver, and consumption due to ongoing coagulation during sepsis (103–105). Intravascular clotting and microvascular thrombosis result in secondary protein C, protein S, and antithrombin III consumption (106). Kudo et al. (107) established four clinical phenotypes based on the severity of coagulopathy characterized by low platelet counts, high levels of FDP and D-dimer, and high organ dysfunction scores in ICU patients. Similarly, the administration of recombinant human-activated protein C inhibits leukocyte-endothelial interaction, suppresses inflammatory cytokine production, and protects the microcirculation by inhibiting plasminogen activator inhibitor-1 modulating fibrinolysis (108, 109). Recently, suppression of fibrinolysis, termed “fibrinolytic shutdown,” has gained attention. An increase in the tissue plasminogen activator due to the significant increase in PAI-1 by endothelial cells results in fibrinolytic shutdown (110). In vivo models of endotoxemia have shown that fibrin deposition in the kidneys or adrenal glands is mainly attributed to plasminogen activators, downregulation of anticoagulant, and fibrinolytic pathways (111, 112). The inflammatory cytokines such as tumor necrosis factor-α (TNF-α), interleukin 1, and the complement system in sepsis models downregulate the anticoagulant pathways in sepsis (62). Thrombin also contributes to fibrinolysis resistance by alerting fibrin structures, making them more compact and less permeable (113). This can promote thrombus formation within small and medium vessels, leading to multiorgan failure. The emerging role of point-of-care coagulation tests has received attention to detect sepsis-induced coagulopathy necessary for prognosis and diagnosis (114).

## THE COMPLEMENT SYSTEM IN SEPSIS

The innate immune system orchestrates a protective response against microbes by multiple players of the innate and adaptive with the complement system. Similar to other defense systems, the complement response becomes excessive and inappropriately stimulated in sepsis, leading to dysregulated and potentially harmful behavior (115, 116). The early stages of sepsis systematically activate the complement system generating a large amount of C3a and C5a (115, 116). Activated complement systems generate a proinflammatory response that increases vascular permeability and chemotaxis of leukocytes (117). The overwhelming activation of complement results in vascular leak and host tissue damage (118). Activated complement with endothelial activation disrupts cellular barriers, consequently forming edema in the brain, lung, and liver (118–121). Complement factors (such as C1q, C3a, and C5a) induce blood-brain barrier damage, increasing complement synthesis and leakage of serum complement proteins into the subarachnoid space of the central nervous system, consequently causing septic encephalopathy (122). However, the complements factors (specifically C3a and C5a) also have neuroregenerative effects apart from being neurotoxic, as they mediate the release of neurotrophins (123, 124). The cells lining the reticuloendothelial system in the liver can clear the complement proteins, protecting hepatocytes from complement-mediated injury; however, excessive activation consumes C5a at the later stages of sepsis, resulting in neutrophil dysfunction with impaired bactericidal activity (88, 125). Complement activation in the liver upregulates adhesion molecules, increasing the recruitment of leukocytes (126, 127). On leukocyte recruitment, C5a activates leukocytes to mount an oxidative burst with NADPH oxidase assembly and chemotactic response for phagocytosis and bacterial clearing (115, 116).

C5a also binds to a second receptor, apart from C5aR, a C5-like receptor (C5L2) (128). The C5L2 and C5aR receptors asynchronously shown to be downregulated on neutrophil’s surface during septic shock correlate to multiorgan failure development (128). The complement system is vital in cardiac malfunction during sepsis. The C5a interacts with C5a receptors on the surface of cardiomyocytes that downregulates Na^+^-K^+^-ATPase, sarco-/endoplasmic reticulum Ca^2+^-ATPase and Na^+^/Ca^2+^ exchanger, resulting in loss of calcium hemostasis, essential to maintain cardiac contractility (129, 130). Therefore, enhanced C5a is associated with impaired cardiac function, whereas the administration of C5a-specific blocking antibody reverses septic cardiomyopathy (131–134). The complement system also plays a vital role in coagulation with the immune system. C5b-9 terminal complex facilitates tissue factor expression by leukocytes, increasing thrombogenicity by simultaneous induction of coagulation (135). They also modify phospholipid membranes required for tissue factor expression (135, 136). Complement factors C5b-9 increase phosphatidylserine expression to provide a catalytic surface for prothrombinase on platelets, cleaving the prothrombin and generating thrombin (137, 138). Hence, the complement system can influence the coagulation pathway and innate response deleterious in sepsis.

## INTERACTION OF ALL DOMAINS IN SEPSIS PATHOPHYSIOLOGY

Originally, it has been believed that sepsis is an inflammatory host response to infection; however, the inflammatory response activates the other systems, such as endothelial, complement, and coagulation. We believe that the pathophysiology of sepsis is complicated and intertwined due to several factors that play a role in the host response and the presenting symptoms of the patient. Some patients present with strong fighting responses, whereas others deteriorate to a dysregulated state of immunity. The endothelium forms a dynamic equilibrium between inflammation, innate immunity, complement, and coagulation to delicate a host response in sepsis (37). The cytokine storm during hyperinflammation damages the endothelium, causing dysregulated vascular tone, and impairing vascular permeability (38–42). Similarly, the endothelium contributes to proinflammation by recruiting inflammatory cells and releasing inflammatory mediators (53). The initial cytokine storm causes fever, shock, respiratory failure, and early death due to multiple organ dysfunction (16, 17). The complement system activation upregulates adhesion molecules increasing the recruitment of leukocytes and chemotactic response for phagocytosis and bacterial clearing (115, 116, 126, 127). The hypercoagulability of sepsis driven by the release of tissue factors from disrupted endothelial cells causes the systemic activation of the coagulation cascade (139). Activated neutrophils adhered to the injured endothelial cells release NETs that facilitate platelet aggregation (94–97). The subsequent activation of the coagulation system increases the production of fibrin-rich microvascular clots in sepsis. The microcirculatory dysfunction leads to loss of smooth muscle tone reactivity and peripheral vasodilation, ensuing organ hypoperfusion, impaired oxygen delivery to the tissues, and anaerobic glycolysis lactic acidosis (67). Therefore, the pathophysiology of sepsis is dysregulated response to infection that triggers cascades of interconnected systems. This cascade involves multiple players and requires detecting different biomarkers depending on the host status for early diagnosis.

## EARLY SEPSIS DIAGNOSIS

The clinical transition from infection to sepsis is termed “time zero.” Early warning scores identify patients at high risk of clinical transition to sepsis and help to recognize critical changes in the patient’s condition; however, little is known about at which stage of sepsis this equilibrium is disrupted (140, 141). This tipping point varies from patient to patient and is likely impacted by host status (such as noncommunicable diseases, injuries, and infections), genetic predisposition, and pathogen type and burden (5, 16, 17, 19, 142, 143). The elderly with chronic comorbidities such as cancer and diabetes are at an increased risk of sepsis due to the functional impairment of cell-mediated immunity and humoral immune responses with age (5, 144). Often, early sepsis manifests as reduced capillary refill time, mottled skin, increased respiratory rate, and altered mental status; hypotension will then ensue, reflecting the onset of circulatory failure, followed by shock, respiratory and renal failure, and premature death due to multiple organ dysfunction (37).

Early resuscitation and antibiotic treatment are essential to reestablish organ perfusion (145). However, some patients fail to respond and exhibit depressed myocardial function and inadequate systemic oxygen delivery, increasing anaerobic glycolysis and lactate production (19, 142). Thus, the clinical manifestation of sepsis varies from patient to patient depending on the ability of the individual’s immune system to prevent and manage infections based on risk factors for sepsis.

The Surviving Sepsis Campaign recommends administrating intravenous broad-spectrum antibiotics within 1 h of blood culture results. Every 1-h delay in antibiotic administration is associated with increased in-hospital mortality among patients with sepsis in ED (146). To the best of our knowledge, there are only 11 studies using biomarkers to diagnose sepsis in the emergency department, and none looked at biomarkers involved in the complex pathophysiology (150–153, 156–158, 160–163). An ideal biomarker (or set of biomarkers) should become abnormal before clinical signs and symptoms develop and possess near-perfect sensitivity and specificity (147). This deficit identifies the poor understanding of sepsis pathophysiology and reflects the need to identify the gaps in the current knowledge on sepsis pathophysiology to diagnose sepsis. Procalcitonin (PCT) has been considered the promising biomarker clinically used to detect sepsis in the ED; still, it has a low positive predictive value and is downregulated in viral infections (148–151). Measuring monocyte distribution width is more accurate than PCT levels in ED sepsis diagnosis using the sepsis-3 definition (152, 153). Presepsin, also referred to as CD14 (cluster-of-differentiation) functions, a receptor for peptidoglycan with PCT, has a comparable performance with diagnosing sepsis in the ED (154). Similar to PCT, presepsin is specific for bacterial infections (especially Gram-negative) and gives false-positive results in certain conditions such as renal failure or burns (155). Lipopolysaccharide-binding protein (LBP), an acute phase protein important in Gram-negative infection, has a diagnostic accuracy lower than that of PCT (156). Similarly, the performance of a proinflammatory mediator that binds and activates neutrophils, known as pancreatic stone protein, and an anti-inflammatory mediator, soluble CD25 (sCD25), has been comparable with PCT (153). Another observational study that used the older sepsis definition, systemic inflammatory response syndrome (SIRS) criteria, found the diagnostic performance of neutrophil gelatinase-associated lipocalin (NGAL) and tissue inhibitor of matrix metalloproteinases-1 (TIMP-1) greater than PCT in the ED (157). However, there have been many conflicting reports on using PCT as a biomarker with others. Interleukin-6 had a superior diagnostic value compared with PCT and even CRP in patients with ED diagnosed with sepsis (158). Neutrophil CD64 expression shows high specificity and positive predictive value in distinguishing sepsis from patients with no sepsis in the emergency department, making it an accurate biomarker for this purpose (162). Newer biomarkers have been studied for their role in early sepsis identification. They have focused on performance with PCT. None considered the complex pathophysiology of sepsis and biomarkers interlinked to each pathway. Most of these studies focused on the inflammation pathway (Table 1). In addition, the lack of progress in the early diagnosis of sepsis using a biomarker is attributed to finding a single most suitable marker. However, the host response in sepsis involves multiple players at various stages during the disease process. Inflammation and coagulation are critical in the host’s responses to infection (159). Given these complexities, future studies focusing on the immunothrombotic markers are important for early sepsis diagnosis.

**Table 1.** Biomarkers for early diagnosis of sepsis

	Author, Yr	Number of Patients	Biomarkers	Biological Domain	Conclusion
1.	Bo et al., 2013 (160)	859	Presepsin compared with PCT	Inflammation	Presepsin is a valuable biomarker for early diagnosis of sepsis, risk stratification, and evaluation of prognosis in patients with sepsis in the ED.
2.	Laura et al., 2014 (151)	513	PCT, CRP, and WBC	Inflammation	CRP and PCT are reliable diagnostic and biomarkers compared with WBC and can be used in combination with severity clinical score.
3.	Miaomiao et al., 2014 (157)	480	NGAL, MMP-9, TIMP-1, and PCT	Inflammation and endothelium	NGAL and TIMP-1 are valuable for early diagnosis of sepsis in the ED.
4.	Caitlin et al., 2014 (150)	66	MRproADM, MRproANP, PCT, copeptin, and proET-1	Endothelium and microcirculation	There were no differences between patients with septic and nonseptic for MRproADM, MRproANP, copeptin, or proET-1. Combination of systemic inflammatory response syndrome (SIRS) criteria and PCT levels is useful for the early detection of sepsis in patients with ED.
5.	*Xin et al., 2015 (161)	1,815	Presepsin	Inflammation	Presepsin exhibits a good diagnostic accuracy for sepsis compared with patients with systemic inflammatory disease.
6.	Toh et al., 2016 (162)	51	sPLA2-IIA and CD64	Inflammation	sPLA2-IIA showed superior performance in sepsis diagnosis compared with CD64. In distinguishing sepsis from nonsepsis groups.
7.	Luis et al., 2017 (153)	152	PCT, PSP, and sCD25 levels	Inflammation	PSP and sCD25 were comparable with PCT to identify patients with sepsis in the ED.
8.	Luis et al., 2018 (156)	49	LBP, CRP, and PCT	Inflammation	In adult patients with ED with suspected infection, the diagnostic accuracy for sepsis of LBP was similar to that of CRP but lower than that of PCT.
9.	Juhyun et al., 2019 (158)	142	IL-6, PTX3, and PCT	Inflammation	The diagnostic value of IL-6 was superior to those of PTX3 and PCT for sepsis and septic shock.
10.	Elliott et al., 2019 (163)	2,158	MDW and WBC count	Inflammation	MDW alone was effective for the early detection of sepsis in the ED.
11.	Pierre et al., 2021 (152)	1,517	MDW, PCT, and CRP	Inflammation	MDW in combination with WBC has the diagnostic accuracy for diagnosis in the emergency department.

*This is a systematic review. CRP, C-reactive protein; MMP-9, matrix metalloproteinase-9; LBP, lipopolysaccharide-binding protein; MDW, monocyte distribution width; MRproADM, midregional proadrenomedullin; MRproANP, midregional proatrial natriuretic peptide; NGAL, neutrophil Gelatinase-Associated lipocalin; PCT, procalcitonin; ProET-1, proendothelin-1; PSP, pancreatic stone protein; PTX3, pentraxin 3; sPLA2-IIA, group II secretory phospholipase A2; TIMP-1, tissue inhibitor of matrix metalloproteinases-1; WBC, white blood cell.

## CONCLUSIONS

The complex interaction between different biological domains in sepsis pathophysiology has been poorly understood. Sepsis has been known as a dysregulated reaction to infection; however, the switch from infection to sepsis involves the inflammation pathway that intersects with the coagulation system for endothelial stimulation and microcirculatory dysfunction. The early diagnosis is often difficult as the heterogeneity in the individual response is huge, and the signs and symptoms of sepsis are nonspecific. The development of validated biomarkers has been impossible due to the focus on inflammatory markers, whereas the pathophysiology involves multiple biological pathways at different levels. We show that immunothrombosis plays an important role in determining the switch from infection to sepsis and is crucial to fill the literature gap in the search for potential biomarkers for early sepsis diagnosis. Future studies using immunothrombosis markers for sepsis will help in determining the optimal duration of treatments, antibiotic stewardship, and novel diagnostic approaches to improve care.

## GRANTS

A.F.-R. received funding from the Canadian Institutes of Health Research for Canadian Sepsis Research Network: Improving Care Before, During and After Sepsis (Grant No. RN391741-430986).

## DISCLOSURES

No other conflicts of interest, financial or otherwise, are declared by the authors.

## AUTHOR CONTRIBUTIONS

J.A. prepared figures; J.A. drafted manuscript; J.A., A.A.M., and A.F.-R. edited and revised manuscript; J.A., A.A.M., and A.F.-R. approved final version of manuscript.
